# Longitudinal association between sleep and Alzheimer's pathology

**DOI:** 10.1002/alz.71228

**Published:** 2026-03-10

**Authors:** Bery Mohammediyan, Andrée‐Ann Baril, Alfonso Fajardo Valdez, Frédéric St‐Onge, Alexa Pichet Binette, Julie Carrier, Maiya R. Geddes, Simon Ducharme, Maxime Montembeault, Jean‐Paul Soucy, John Breitner, Judes Poirier, Sylvia Villeneuve, for the PREVENT‐AD Research Group

**Affiliations:** ^1^ Center for Advanced Research in Sleep Medicine Research Center of the CIUSSS‐NIM Montreal Quebec Canada; ^2^ Department of Medicine Université de Montréal Montreal Quebec Canada; ^3^ Douglas Mental Health University Institute Montreal Quebec Canada; ^4^ Centre de Recherche de l'Institut Universitaire de Gériatrie de Montréal Montréal Quebec Canada; ^5^ Department of Physiology and Pharmacology Université de Montréal Montréal Quebec Canada; ^6^ Clinical Memory Research Unit Department of Clinical Sciences Malmö Lund University Lund Sweden; ^7^ Department of Psychology University of Montreal Montreal Quebec Canada; ^8^ Department of Psychiatry Faculty of Medicine McGill University Montreal Quebec Canada; ^9^ Massachusetts Institute of Technology Cambridge Massachusetts USA; ^10^ McConnell Brain Imaging Centre Montreal Neurological Institute Montreal Quebec Canada

**Keywords:** amyloid, circadian rhythms, PET, sleep, tau

## Abstract

**INTRODUCTION:**

Since sleep disturbance is a modifiable risk factor for Alzheimer's disease (AD), we tested associations between sleep and AD pathology in cognitively unimpaired (CU) persons.

**METHODS:**

We included 223 participants from the PREVENT‐AD cohort with self‐reported measures of sleep, objective actigraphy measures of sleep, and positron emission tomography (PET) scans for AD pathology quantification. Repeated PET scans (mean follow‐up: 4.31 ± 0.55 years) were available for 103 participants. We conducted robust linear models (RLM) for cross‐sectional analyses and RLMs using the annual change in AD pathology for longitudinal analyses.

**RESULTS:**

All actigraphy‐based sleep variability measures were associated with tau burden (duration: *β* = 0.121 [95% confidence interval {CI} = 0.010; 0.232], *p* = 0.034; efficiency: 0.122 [0.010; 0.235], 0.033; fragmentation: 0.115 [0.010; 0.221], 0.033). Greater variability in sleep fragmentation was also associated with amyloid burden (0.074 [0.008; 0.140], 0.028), and variability in sleep efficiency portended amyloid burden and faster accumulation over time (0.075 [0.009; 0.141], 0.026; 0.164 [0.008; 0.320], 0.039; respectively).

**DISCUSSION:**

Irregularity in sleep patterns is associated with higher pathological burden and faster amyloid accumulation.

## Highlights

- Greater day‐to‐day variability in actigraphy‐based sleep measures was associated with higher amyloid and tau burden in cognitively unimpaired older adults at risk for Alzheimer's disease (AD).
- Greater day‐to‐day sleep variability—particularly variability in sleep efficiency—was also associated with faster amyloid accumulation over time.
- Sleep variability may serve as an early and sensitive marker of preclinical AD pathophysiology, highlighting its potential as a target for preventive interventions.

## 1 BACKGROUND

Brain aggregation of amyloid beta (Aβ) and tau proteins is a pathological hallmark of Alzheimer's disease (AD).^1^ Such aggregation begins when individuals are cognitively unimpaired (CU), and this presymptomatic phase of AD offers a time window for preventive interventions before significant atrophic changes or cognitive impairment occur.^2^ The modification of risk factors, potentially delaying Aβ and tau accumulation and symptom onset in CU individuals, may help mitigate the current AD epidemic. The 2024 Lancet Commission Report on Dementia Prevention, Intervention and Care suggested that modification of 14 risk factors could prevent or delay almost 45% of dementia cases.^3^ An additional modifiable factor is sleep disturbance in late life, which is hypothesized to account for 5.7% of dementia risk.^4^ Sleep disturbances affect about 50% of individuals aged 65 years and older, and the severity of sleep symptoms increases with AD symptoms, making it a promising target for preventive trials.^5^, ^6^, ^7^, ^8^, ^9^, ^10^

While several studies have suggested that sleep disturbances can influence cognitive performance and increase the risk of mild cognitive impairment (MCI) and dementia, it is less clear whether sleep disturbances are associated with AD pathology.^11^, ^12^, ^13^ The few cross‐sectional studies available among CU individuals suggest that worse sleep quality and increased intra‐individual variability in sleep patterns across nights are associated with higher amyloid and tau burden measured in cerebrospinal fluid (CSF) and positron emission tomography (PET).^12^, ^13^, ^14^, ^15^, ^16^, ^17^ Moreover, studies assessing sleep using polysomnography, the gold standard for measurement of sleep architecture, found that a reduction in slow‐wave sleep was associated with higher levels of amyloid and tau in older adults.^18^, ^19^ Animal models also suggest that sleep deprivation exacerbates amyloid and tau burden.^20^, ^21^ One study found that mice had higher glymphatic clearance of amyloid and tau in the interstitial fluid and in the CSF when sleeping compared with conditions of wakefulness or sleep deprivation.^21^ Improving circadian rhythm and sleep architecture has also been linked to lower Aβ accumulation, suggesting that reducing sleep disturbances may be a target for AD prevention.^22^

To better understand how sleep patterns associate with AD pathology in aging, we tested associations between sleep (subjective and objective measures) and cross‐sectional and longitudinal measures of AD pathology in a cohort of older adults who were CU at the time of their sleep measurement. Members of this PResymptomatic EValuation of Experimental or Novel Treatments for Alzheimer's Disease (PREVENT‐AD) cohort are at higher risk of developing AD due to a first‐degree familial history of late‐onset clinical AD. Subjective sleep quality was assessed using the Pittsburgh Sleep Quality Index (PSQI). Objective sleep was assessed with actigraphy over 7 consecutive days, focusing on variations in sleep patterns from day to day. The actigraphy studies were motivated by the observation that sleep variability has been suggested as an early marker of neurodegenerative processes.^12^, ^16^, ^23^, ^24^ For comparison with other studies, supplementary analyses also tested associations between sleep patterns averaged over 7 days of actigraphy and AD pathology.

## 2 METHODS

### 2.1 PREVENT‐AD cohort

PREVENT‐AD is an ongoing investigator‐driven longitudinal study created in 2011 and includes 387 older adults with a parental or multiple‐sibling history of sporadic AD. Participants were eligible for enrollment if they were 60 years of age or older, or 55–59 years if within 15 years of the age of onset of their youngest‐affected relative.^25^, ^26^, ^28^ Normal cognition at enrollment was required, demonstrated by a brief cognitive screening at study entry using the Clinical Dementia Rating (CDR) and Montreal Cognitive Assessment (MoCA). In a few cases of ambiguous CDR (0.5) or MoCA (≤26), participants were evaluated by a certified neuropsychologist using an extensive neuropsychological battery to confirm normal cognition. Participants were then followed annually with core measurements including clinical evaluation and cognition.

**RESEARCH IN CONTEXT**

1. **Systematic review**: We reviewed the literature using PubMed and Google Scholar. Prior studies have suggested that poor sleep is associated with cognitive decline and Alzheimer's disease (AD) pathology. However, findings in the preclinical phase of the disease are inconsistent, and longitudinal studies remain limited.
2. **Interpretation**: In this longitudinal study of older adults with a family history of sporadic AD, objective actigraphy‐based day‐to‐day sleep variability was associated with greater amyloid and tau burden cross‐sectionally, and with faster amyloid accumulation over time. Subjective sleep quality was related to tau‐positron emission tomography (PET) only in a subsample of individuals who completed their sleep assessment within 1 year of their PET scan. Moreover, among all average sleep measures tested only average sleep duration was associated with amyloid pathology. These findings suggest that sleep variability, more specifically sleep efficiency and sleep fragmentation variability, may be a more sensitive early marker of AD‐related changes than average sleep measures or self‐reported sleep quality.
3. **Future directions**: Future studies should examine mechanistic pathways linking sleep variability to AD pathogenesis, including roles of glymphatic clearance, circadian regulation, and comorbid conditions. Incorporation of polysomnography and interventional approaches targeting sleep regularity will help determine whether modifying sleep variability can alter AD trajectory.

**FIGURE 1:**
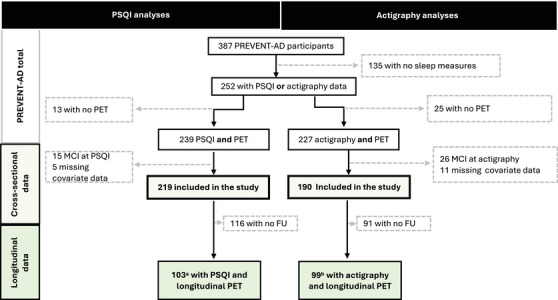
Flow chart of participants included in the study. Two hundred twenty‐three participants were included in the study: 219 PREVENT‐AD participants answered a self‐reported sleep questionnaire (PSQI) at baseline, and 190 completed 7 days of actigraphy data collection. Most of the participants (*n* = 186) had both PSQI and actigraphy data. ^a^Longitudinal tau pathology available for 97 participants. ^b^Longitudinal tau pathology available for 93 participants. FU, follow‐up; MCI, mild cognitive impairment; PET, positron emission tomography; PREVENT‐AD, PRe‐symptomatic EValuation of Experimental or Novel Treatments for Alzheimer's Disease; PSQI, Pittsburgh Sleep Quality Index.

We studied 223 PREVENT‐AD participants who had available sleep (PSQI or actigraphy), PET (amyloid or tau), and cognitive data. The last included a demonstration that they were CU when sleep data were collected (Figure 1 for the flow chart of the participants included in the study).^26^, ^28^ To be included, participants also needed to have available data on age, sex, and body mass index (BMI; weight in kilograms divided by height in meters squared). The PSQI and the actigraphy data were not always collected simultaneously. Participants who were CU at the time of their PSQI assessment but impaired at the time of their actigraphy assessment were only included in the PSQI analyses. Thus, among the 223 participants, 219 were included in the PSQI analyses, 190 in the actigraphy analyses, and 186 in both sets of analyses.

The cohort has been tested annually, with cognition being tested yearly, whereas other measures, such as actigraphy and PET, were tested at selected years.^26^, ^28^ Cross‐sectional analyses included baseline sleep measurements and the most proximate (before or after) amyloid and tau‐PET scan. Some 103 participants had second amyloid and tau scans performed about 4 years later (mean follow‐up: 4.31 ± 0.55 years, range: 1.59–6.11 years). Longitudinal analyses included baseline sleep measurements and the amyloid and tau‐PET annual change, which was derived from the rate of change between the first and second PET scan.

All participants provided informed consent. PREVENT‐AD has approval from the McGill Institutional Review Board and/or Douglas Mental Health University Institute Research Ethics Board, conformed with the ethical principles of the Declaration of Helsinki.

### 2.2 PSQI questionnaire

Subjective sleep quality was evaluated using the PSQI, a self‐administered questionnaire that evaluates sleep quality over the previous month to provide a global score (0–21), where higher scores indicate poorer sleep quality.^29^ In cross‐sectional analyses, the mean time difference between the PSQI and the most proximate PET scan was 2.56 ± 2.01 years, range: 0.00–6.97 years. If the PSQI was assessed more than once, the first assessment was used in the analyses.

### 2.3 Actigraphy protocol and processing

Objective sleep was measured using actigraphy. The mean time difference between the first actigraphy measure and the most proximate PET scan was 0.68 ± 1.21 years, range: 0.00–4.79 years. When actigraphy was assessed more than once, the first assessment was used in the analyses.

The actigraphy data were collected over an interval of 1 week using a wrist Actiwatch (Philips Respironics, PA, USA). In parallel, participants completed a sleep journal including information on sleep/wake routine. Ninety‐eight percent of participants wore the watch for at least six consecutive nights (mean nights: 7.08 ± 0.62). Actigraphy data (in activity counts of movement over a given time period) were processed through the Actiware software (version 6.0) with a medium threshold for sensitivity of 40 activity counts/per minute.^30^ Data were collected using 15‐second epochs. Time in bed, as detected by the Actiware algorithm, was corroborated by two raters based on the sleep journals (the raters’ concordance was 94%). The Actiwatch allows estimation of a wide variety of sleep variables. Hypothesis‐driven analyses used day‐to‐day variability (standard deviation) of (1) sleep duration (minutes); (2) sleep efficiency (%, sleep duration/time in bed); and (3) sleep fragmentation index (percent of mobile + immobile < 1 minute bouts over the sleep period, representing the proportion of the sleep period classified as restlessness).^16^, ^23^ Results related to the average (predictor) actigraphy measures are reported in Supporting Information for comparison purposes.

### 2.4 Amyloid and tau PET acquisition and processing

We used [^18^F]NAV4694 as a tracer to quantify the presence of amyloid beta, using a scanning window 40–70 minutes after injection of approximately 220 MBq (6 mCi). Six frames of 5 min each were acquired. To quantify the presence of tau, we used ^18^F‐flortaucipir as a tracer and a scanning window 80–100 min after injection of approximately 370 MBq (10 mCi). Four frames of 5 minutes each were acquired. PET images were reconstructed by applying a three‐dimensional (3D) ordinary Poisson ordered subset expectation maximum ([OP‐OSEM], 10 iterations, 16 subsets) algorithm. Decay and motion correction were applied to the images, and scatter correction was performed using a 3D scatter estimation method.^31^ All PET scans were performed at the McConnell Brain Imaging Centre at the Montreal Neurological Institute (Montreal, Quebec, Canada) on a brain‐dedicated CTI/Siemens high‐resolution research tomograph (HRRT) scanner. Most amyloid and tau scans were acquired on 2 consecutive days, between 2017 and 2019 for baseline and 2021 and 2023 for follow‐ups. Each participant also underwent structural magnetic resonance imaging (MRI) scans, performed at the Cerebral Imaging Centre of the Douglas Mental Health University Institute (Montreal, Quebec, Canada). MRI scans acquired before 2019 were performed using a 3T Siemens TIM Trio scanner (Siemens, Munich, Germany) with parameters previously described.^26^, ^28^ Scans acquired after 2019 used a 3T Siemens Prisma Fit scanner (Siemens Medical Solutions, Erlangen, Germany) with parameters previously described.^26^, ^28^

Preprocessing of PET images was performed using an in‐house standard pipeline (github.com/villeneuvelab/vlpp). In brief, PET image frames were realigned, averaged and then co‐registered to the temporally closest T1‐weighted MRI image, which was processed and divided into 34 bilateral regions of interest (ROIs) based on the Desikan–Killiany atlas using FreeSurfer (version 5.3). After masking PET images to eliminate the CSF signal, the images underwent smoothing using a 6 mm Gaussian kernel. Standardized uptake value ratio (SUVR) was calculated as the mean tracer uptake in the region of interest divided by the mean uptake in cerebellar gray matter and inferior cerebellar gray for amyloid and tau, respectively.^32^, ^33^ A global amyloid index that included frontal, temporal, parietal, and posterior cingulate cortex was used for statistical analysis.^32^ The tau temporal meta‐ROI, including bilateral entorhinal, amygdala, parahippocampal, fusiform, inferior and middle temporal cortices, was used for statistical analysis.^34^

For visualization purposes we also repeated the analyses at a voxel‐wise level using Statistical Parametric Mapping (SPM12) in a gray matter mask.^35^ To assess longitudinal changes, we registered SUVR maps in the Montreal Neurological Institute (MNI) 152 template. We then subtracted the baseline SUVR maps from the follow‐up SUVR maps and divided the result by the time difference between the baseline and follow‐up.

In supplementary longitudinal analyses we further divided participants into amyloid‐positive (A+) and amyloid‐negative (A–) using a threshold of 1.26 SUVR.^36^ This value was an average of a liberal threshold (2 standard deviations [SD] above the mean from a young adult sample of 11 individuals) and a conservative one (established using Gaussian mixture modeling).

### 2.5 Statistical analyses

We used robust linear regression (RLM) models to evaluate cross‐sectional associations between sleep variables (PSQI, actigraphy day‐to‐day sleep variability) and both amyloid and tau abnormality.^37^ To test for longitudinal associations in individuals having two PET scans, we estimated each individual's annual change in amyloid and tau SUVRs. We then used these slopes as outcome variables in RLM models, testing association with baseline subjective or objective sleep measures. RLM has the advantage of being less influenced by outliers.^38^ Standardized coefficient (β) and standardized confidence interval (CI) are reported here, with values between 0.1 and 0.3 being considered small effects, while values between 0.3 and 0.5 were taken as medium effects and values >0.5 as large effects.^39^ Because the time difference between PSQI and PET often exceeded 1 year, we also conducted a sensitivity analysis that included only individuals whose PSQI assessment was within 1 year of their PET scan (*n* = 68).

In supplementary analyses, we tested the association between averaged actigraphy sleep variables and AD using RLM models. We also repeated longitudinal analyses with baseline amyloid status (A+/A–) as an interactive term, given that A+ individuals accumulate Aβ faster than A– individuals. Finally, we repeated all analyses using apolipoprotein E (*APOE*) status (genotype including *APOE* ε4 vs. others without) and self‐reported sex (female/male) as interactive terms given previous literature suggesting that these variables are associated with both sleep quality and AD pathology.^3^, ^27^, ^40^

Otherwise, models were adjusted for age at baseline, sex, and BMI, a prominent risk factor for obstructive sleep apnea.^3^, ^41^ Other adjustments included time between predictor (PSQI or actigraphy sleep measure) and PET outcome (both amyloid and tau; cross‐sectional analyses only). All analyses addressed the same overarching hypothesis‐driven research question, and the sleep measures were highly correlated (see Pearson correlation coefficient matrix in Supporting Information, Figure S1). Accordingly, we used a two‐sided significance criterion of *p* ≤ 0.05 to identify significant associations. In interpreting the results, we nevertheless considered the full pattern of tested associations, with isolated findings interpreted more cautiously than associations consistently observed across multiple sleep measures. As an additional, more conservative criterion, we also report which results would survive Benjamini–Hochberg false discovery rate (FDR) correction, applied separately for amyloid and tau outcomes.

For amyloid and tau results at the voxel‐wise level, we report both uncorrected results with a *p* < 0.0001 in a minimum cluster of 200 voxels and family‐wise error (FWE) correction.

## 3 RESULTS

### 3.1 Participants

The full sample was 70.93% female, and 39.21% had the *APOE* ε4 allele. Participants’ mean age was 68.16 years (SD = 5.09; range: 58.59–83.62) at the time of their baseline PET. Full demographic characteristics can be found in Tables 1 and S1.

**TABLE 1:** Sample demographics.

	PSQI sample (*n* = 219)	Actigraphy sample (*n* = 190)
Age (years)	68.16 (5.09)	68.25 (5.26)
Sex (% female)	161 (70.93)	144 (72.36)
Education (years)	15.54 (3.19)	15.32 (3.24)
BMI (kg/m^2^)	26.74 (4.70)	26.88 (5.07)
Retirement (%)	143 (63)	130 (65.33)
APOE status (% positive)	89 (39.21)	75 (37.69)
**Sleep characteristics (PSQI)**		
PSQI baseline	6.07 (3.42)	6.01 (3.50)^a^
**Time between measures**		
Time between first sleep measure and closest PET (years)	2.56 (2.01)	0.68 (1.21)
Time between first sleep measure and last PET (years)	4.98 (1.22)	4.15 (0.81)
Follow‐up PET scans (years)	4.31 (0.55)^b^	4.35 (0.46)^c^
**Baseline sleep characteristics (average; actigraphy)**		
Sleep duration (min)	436.26 (50.26)	436.17 (49.54)
Sleep efficiency (%)	86.81 (5.73)	86.86 (5.78)
Sleep fragmentation	12.02 (4.74)	11.99 (4.79)
**Baseline sleep characteristics (day‐to‐day variability; actigraphy)**		
Sleep duration (min)	56.19 (26.66)	55.72 (26.31)
Sleep efficiency (%)	5.40 (4.07)	5.32 (4.01)
Sleep fragmentation	4.12 (1.97)	4.11 (1.97)
**AD pathology**		
Amyloid	1.32 (0.30)	1.31 (0.29)
Tau	1.15 (0.12)	1.15 (0.11)

*Note*: Baseline and longitudinal data are presented as *n* (%) for categorical variables, and as mean ± SD for continuous variables. Age, education, BMI, and retirement status are shown at baseline. Two hundred twenty‐three participants were included in the study among whom 186 had both PSQI and actigraphy data. As a result, the demographic, pathological and sleep characteristics of both subsamples were quite similar. Amyloid and tau data represent the mean standardized uptake value ratio (SUVR) at baseline.

Abbreviations: AD, Alzheimer's disease; APOE, apolipoprotein E; BMI, body mass index; PSQI, Pittsburgh Sleep Quality Index; SD, standard deviation.

^a^ PSQI data were available for 186 participants with actigraphy data.

^b^ Longitudinal amyloid and/or tau‐PET was available for 103 participants.

^c^ Longitudinal amyloid and/or tau‐PET was available for 99 participants.

### 3.2 Subjective and objective sleep measures and cross‐sectional and longitudinal AD pathology

**FIGURE 2:**
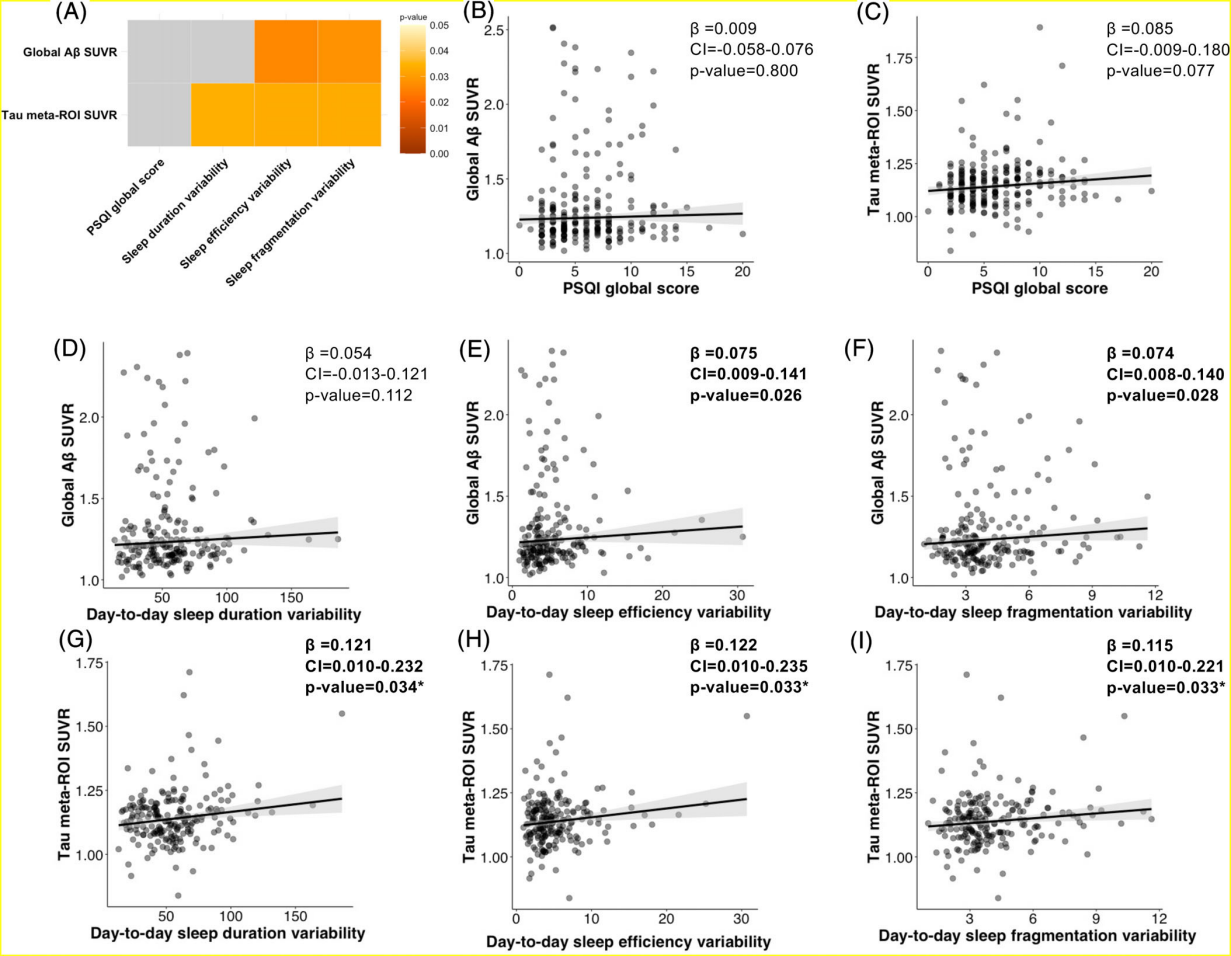
Associations between subjective sleep and sleep variability with baseline AD pathology measured with PET. Age at baseline PET scan, sex, BMI and time between predictor (PSQI or sleep variability) and the outcome (amyloid or tau‐PET) were accounted for in every model. (A) The matrix shows the association between a specified sleep variable (PSQI global score or standard deviation [variability] extracted from 7‐day actigraphy data collection, respectively) and baseline AD PET. The matrix's color grading shows significant associations where lighter colors are more significant and darker colors are closer to *p _uncorrected_* = 0.05. Gray squares show non‐significant associations. (B–I) show the specific scatterplots of these associations. Gray shading represents the 95% confidence interval. Results in **bold** are significant p_uncorrected_ < 0.05. Results with a star (*) survive FDR corrections. Aβ, amyloid‐beta; AD, Alzheimer's disease; β, standardized coefficient; BMI, body mass index; CI, standardized confidence interval; FDR, false discovery rate; PET, positron emission tomography; PSQI, Pittsburgh Sleep Quality Index; ROI, region of interest; SUVR, standardized uptake value ratio.

Figure 2A shows a summary of the cross‐sectional associations between sleep measures and amyloid and tau pathology, controlling for age, sex, BMI, and time between outcome (AD biomarker pathology) and the predictor (PSQI or sleep variability), with stars representing associations surviving FDR correction. Greater variability in day‐to‐day sleep efficiency and day‐to‐day fragmentation index were associated with higher levels of amyloid (*β* = 0.075 [95% CI = 0.009; 0.141], *p* = 0.026, Figure 2E; 0.074 [0.008; 0.140], *p* = 0.028, Figure 2F). Furthermore, greater day‐to‐day variability in sleep duration, sleep efficiency, and fragmentation index was associated with higher levels of tau (0.121 [0.010; 0.232], *p* = 0.034, Figure 2G; 0.122 [0.010; 0.235], *p* = 0.033, Figure 2H; 0.115 [0.010; 0.221], *p* = 0.033, Figure 2I; respectively). When we apply FDR corrections all associations with tau levels remained significant (*p* = 0.045; *p* = 0.045; *p* = 0.045, respectively). We found no cross‐sectional association between PSQI and amyloid or tau pathology (Figure 2B,C). However, in supplementary analyses, we found that increased PSQI global score was associated with higher levels of tau meta‐ROI SUVR when we restricted our participants to individuals who had their PSQI assessment within 1 year of their PET scan (0.180 [0.020; 0.341], *p* = 0.029, Figure S2), an association that did not survive FDR correction.

#### 3.2.1 Longitudinal analyses

**FIGURE 3:**
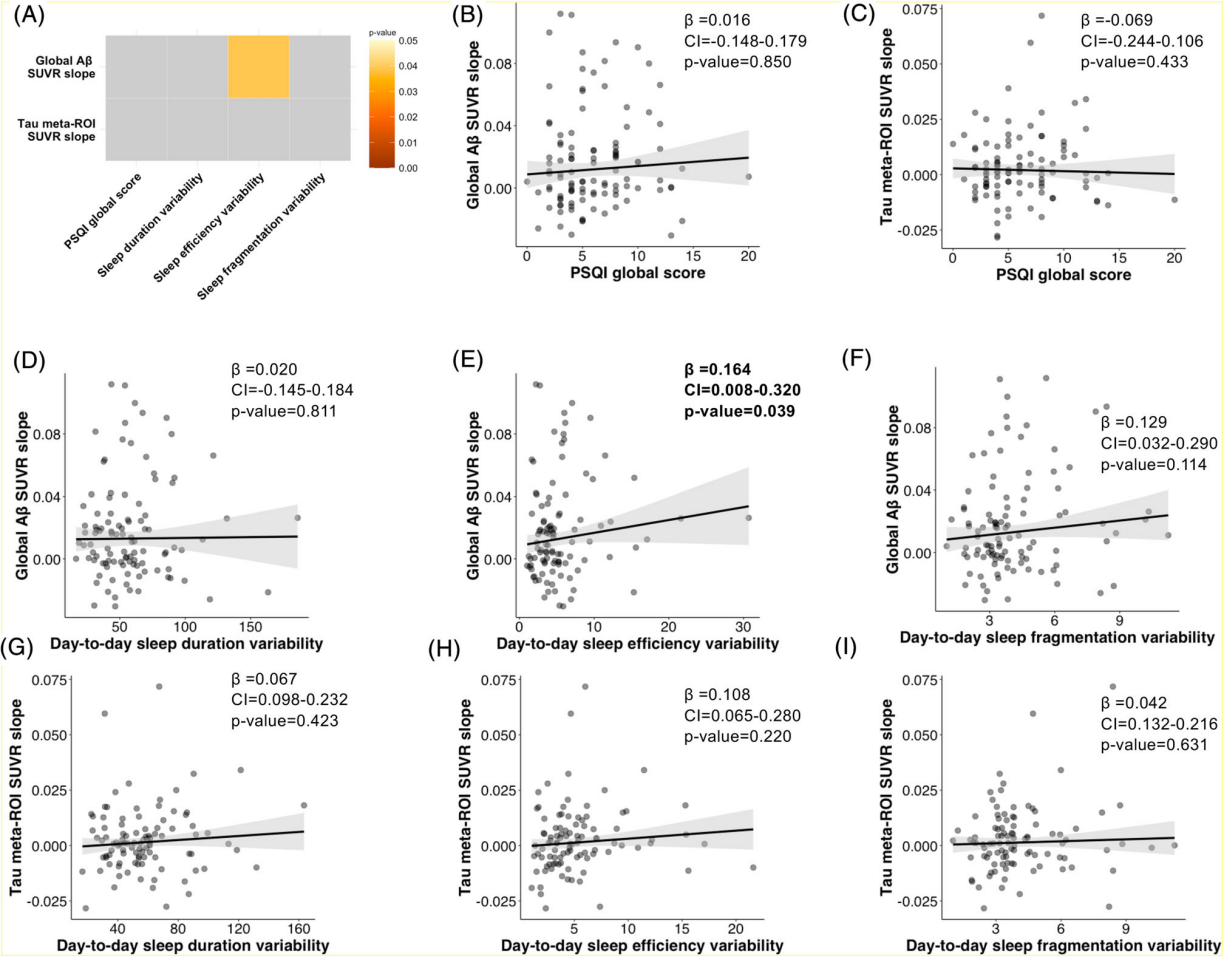
Associations between subjective sleep and sleep variability and longitudinal AD pathology measured with PET. Age at baseline PET scan, sex, and BMI were accounted for in every model. (A) The matrix shows the association between a specified sleep variable (PSQI global score or standard deviation [variability] extracted from 7‐day actigraphy data collection, respectively) and longitudinal AD PET. The matrix's color grading shows significant associations where lighter colors are more significant and darker colors are closer to p_uncorrected_ = 0.05. Gray squares show non‐significant associations. (B–I) show the specific scatterplots of these associations. In all models, longitudinal AD pathology is measured by extracting the annual slope of SUVR accumulation to measure amyloid and tau accumulation, respectively. In all the graphs, gray shading represents the 95% confidence interval. Results in **bold** are significant p_uncorrected_ < 0.05. Results with a star (*) survive FDR corrections. Aβ, amyloid‐beta; AD, Alzheimer's disease; β, standardized coefficient; CI, standardized confidence interval; FDR, false discovery rate; PET, positron emission tomography; PSQI, Pittsburgh Sleep Quality Index; ROI, region of interest; SUVR, standardized uptake value ratio.

Figure 3A shows a summary of the longitudinal associations between sleep measures with amyloid and tau pathology when controlling for age, sex, and BMI. We observed that greater baseline day‐to‐day sleep efficiency variability was associated with faster annual amyloid change (0.164 [0.008; 0.320], *p* = 0.039, Figure 3E), an association that did not survive FDR correction. No other association was significant.

#### 3.2.2 Voxel‐wise analyses

**FIGURE 4:**
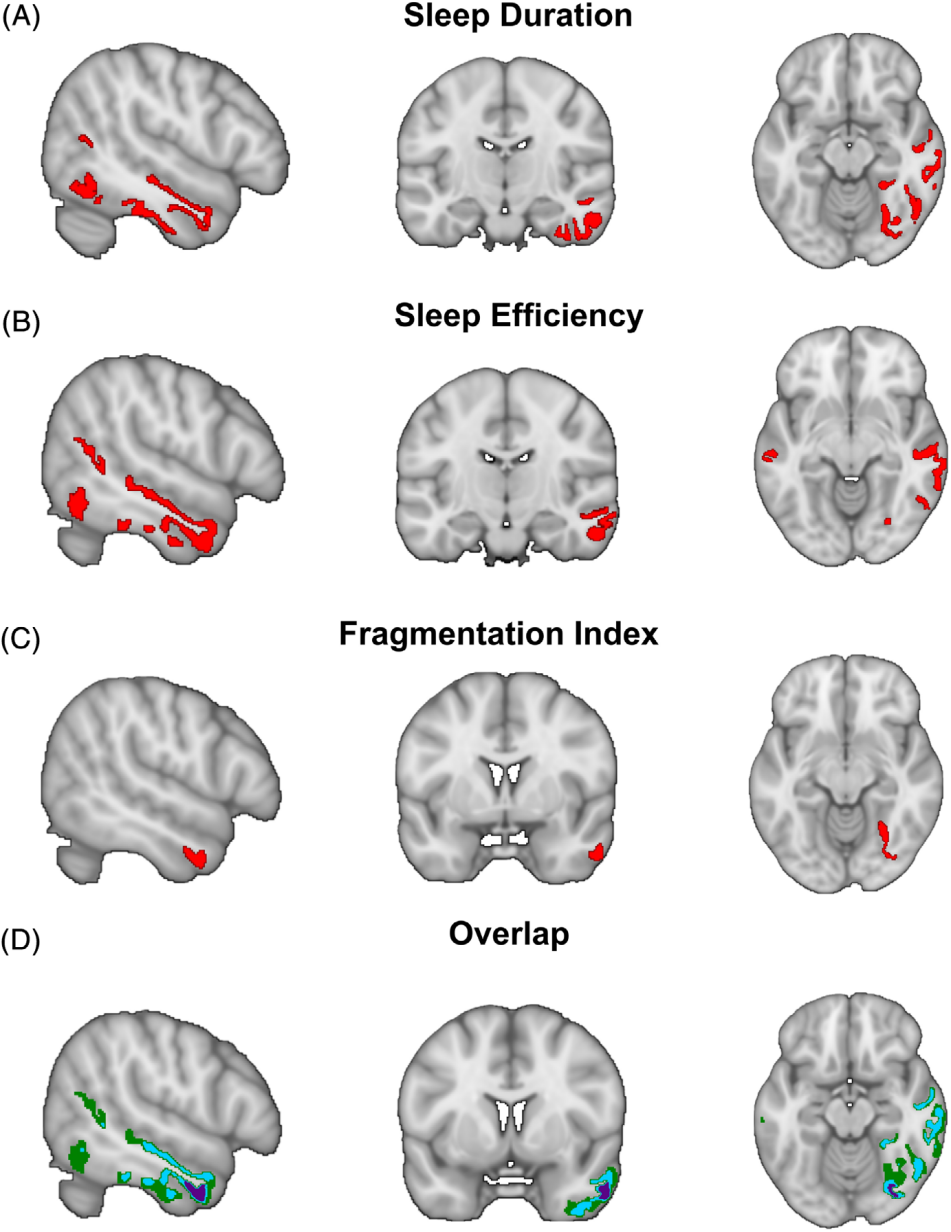
Voxel‐wise significance maps of the association between day‐to‐day sleep variability measures and cross‐sectional tau PET in cognitively unimpaired older adults. Age at PET scans, sex and BMI were accounted for in every model. Panels A–D include 190 participants. In Panels A–C, red shading shows significant clusters at a *p* = 0.0001 with a minimum of 200 voxels. In (D), green represents no overlap (only one variable is associated with these clusters), blue represents overlap of two variables and purple represents overlap of three or four variables. At the voxel level, associations between PSQI and tau‐PET, specified sleep measures and amyloid, and specified sleep measures and longitudinal AD pathology were not significant (not shown). (A) associations between day‐to‐day sleep duration variability and tau‐PET were in the middle and inferior temporal lobe, (B) associations between day‐to‐day sleep efficiency variability and tau‐PET were located focally in the middle and inferior temporal lobe, (C) associations between day‐to‐day fragmentation index variability and tau‐PET were in the inferior temporal lobe, and (D) the overlapping significant voxels between the sleep measures and tau‐PET were spread out in the temporal lobe. BMI, body mass index; PET, positron emission tomography; PSQI, Pittsburgh Sleep Quality Index.

Cross‐sectional voxel‐wise analyses suggested associations between several sleep variability measurements and tau‐PET binding, mainly overlapping in the lateral middle temporal gyrus (Figure 4). No voxel‐wise associations were found with amyloid‐PET or between sleep measures and change in amyloid or tau, and no voxel‐wise level survived FWE correction.

### 3.3 Supplementary analyses

Among the average measures assessed (Figures S3 and S4), we found that higher average sleep duration was associated with lower amyloid levels (−0.034 [−0.146; −0.010], *p* = 0.025, Figure S3B), an association that was no longer significant after FDR correction (*p* = 0.077).

When baseline amyloid status was included as an interaction term in the longitudinal analyses, new associations emerged between actigraphy measures and longitudinal amyloid but not with tau. First, an association was found between day‐to‐day fragmentation variability and longitudinal amyloid accumulation, where A+ individuals with higher day‐to‐day fragmentation variability were accumulating amyloid at a faster rate (0.587 [0.241; 0.933], *p* = 0.001, Figure S5E) when compared to A– individuals. A second interaction was found with average sleep duration, where A+ individuals with lower average sleep duration were accumulating amyloid at a faster rate when compared to A– individuals (−0.900 [−1.329; −0.470], *p* < 0.001, Figure S6A). Both interactions survived FDR correction.

Adding sex and *APOE* status as interaction terms had minimal impact on the results (Figures S7–S14).

## 4 DISCUSSION

In a longitudinal cohort of 223 CU older adults with a first‐degree familial history of AD, we found small but consistent associations between several sleep measures and Alzheimer's pathology. In cross‐sectional associations, these measures were more closely related to tau, but in longitudinal analyses, they related more closely to amyloid. In cross‐sectional analyses, variability in day‐to‐day sleep efficiency was associated with both amyloid and tau burden, and this same variability also portended faster accumulation of amyloid over time, suggesting that day‐to‐day sleep efficiency is a sensitive measure to detect associations with AD pathology.

Others have noted that disturbances of sleep and circadian rhythms, which occur in about half of individuals suffering from AD dementia, are often associated with cognitive decline, and AD pathology.^5^, ^42^, ^43^ Abnormally low or high sleep duration, as well as increased sleep fragmentation and day‐to‐day sleep variability in several sleep parameters, have all been associated with an increased risk of subsequent cognitive impairment.^16^, ^44^, ^45^ A recent longitudinal study in the Rush Memory and Aging Project reported that older adults with higher day‐to‐day sleep variability had an increased risk of AD dementia.^42^ Experimental rodent studies have demonstrated that sleep deprivation and orexin modulations influence brain interstitial fluid levels of amyloid, suggesting that the relationship between the sleep‐wake cycle and AD pathology is causal.^20^, ^21^, ^46^ One human study conducted over a 36‐hour interval similarly observed that an orexin antagonist used for the treatment of insomnia acutely decreased amyloid and tau levels in CSF.^47^ Furthermore, one night of sleep deprivation has been shown to decrease CSF amyloid‐β_42_ levels, a proxy of high brain amyloid.^48^ Rodent studies also suggested that sleep disturbances start when brain amyloid starts to develop.^49^ These observations raise the obvious question of whether sleep abnormality is a consequence or a cause of AD pathology.^50^

In our studies of CU individuals at increased risk of AD, we found that baseline day‐to‐day variability in sleep efficiency, fragmentation and duration measured with actigraphy were all associated with higher levels of tau‐PET. Baseline day‐to‐day variability in sleep efficiency and fragmentation was also associated with amyloid burden. Subjective sleep quality as measured with the PSQI was associated with a small effect on tau, but only among individuals who answered the PSQI within 1 year of their PET scan. In contrast, among all sleep average measures tested, only lower average sleep duration was associated with higher amyloid levels. All effects were of small magnitude, with standardized beta coefficients ranging from 0.1 to 0.2. Worse variability in sleep quality is therefore reliably associated with AD pathology, but the effect is modest. These results also suggest that day‐to‐day variability in sleep parameters, particularly variability in sleep efficiency and fragmentation, is better correlated to AD pathology than the mean of these same parameters in the asymptomatic phase of the disease.

Longitudinal analyses showed associations with amyloid, but not with tau protein. Among all variables, daily variability in sleep efficiency was associated with a more rapid accumulation of amyloid. This isolated finding might simply be a false positive, or it might reflect previously noted baseline associations, given that daily variability in sleep efficiency has been associated with amyloid burden in cross‐sectional analyses and that A+ individuals accumulate amyloid more rapidly. In supplementary analyses, we therefore included baseline amyloid status (A+ vs. A–) as an interaction term and observed no interaction between daily variability in sleep efficiency and baseline amyloid status on amyloid slope, but found interactions with sleep fragmentation variability and average sleep duration, suggesting that associations between these sleep variables and the rate of amyloid accumulation are only detectable among A+ individuals. The association between sleep and amyloid accumulation, therefore, is complex and needs further investigation. With reference to existing literature, we suggest that sleep probably plays a weak role in amyloid accumulation that does not compare to other factors such as *APOE* status.^27^ Mechanisms by which sleep might influence amyloid accumulation include efficiency of the glymphatic system.^21^

Day‐to‐day variability in sleep parameters is increasingly recognized as important in several chronic conditions, often exceeding the significance of mean levels of sleep disturbance.^12^, ^16^, ^51^, ^52^ We previously reported a study on 203 PREVENT‐AD participants showing that day‐to‐day sleep variability was associated with higher ratios of CSF p‐tau181/amyloid‐β_42_ and higher plasma p‐tau231/Aβ_42_.^16^ While sleep variations from day‐to‐day are not well understood, they probably represent a vulnerability that precedes a major shift in sleep behavior. Early in the disease process, AD pathology might disturb the regularity of the sleep pattern or vice versa. A possible mechanism is that early AD pathology exacerbates degradation of subcortical brain regions such as the suprachiasmatic nucleus and/or the locus coeruleus involved in controlling circadian rhythms, causing sleep variability.^53^, ^54^ Other diseases, such as cardiovascular diseases, are also associated with sleep irregularities. These other diseases could also influence the associations between sleep variability and AD pathology, such that sleep would not influence AD pathology directly, but instead be associated with health conditions (e.g., vascular diseases) that promote AD pathology.^55^

Strengths of this study are its large sample of initially CU individuals at increased risk of AD, the inclusion of both subjective and objective measures of sleep, and a PET follow‐up that spanned more than 4 years. Also notable was that 65% of our sample were retired at the time of their baseline sleep measure, thus perhaps mitigating the threat that their sleep patterns reflected daily work activities and constraints instead of natural sleep patterns. Furthermore, previous work had assessed whether retirement had an effect on the association between sleep and AD biomarkers in CSF and plasma, observing none.^16^

We also note several limitations. A limitation is the use of a self‐reported sleep measure and indirect measures of sleep such as actigraphy, while direct measures of sleep, such as polysomnography, represent the gold standard for sleep measures. Future studies might incorporate polysomnography data to better understand changes in sleep architecture as a correlate of AD pathogenesis before the onset of cognitive symptoms. Moreover, we did not specifically assess participants’ psychiatric or long‐standing sleep disorders, medication usage, nor vascular risk factors, all of which would likely affect both subjective and objective sleep quality.^55^, ^56^, ^57^ Furthermore, the generalizability of our findings to other ethnic groups can be questioned because almost all PREVENT‐AD participants are of non‐Hispanic White ethnicity. Finally, although portions of our design were longitudinal in nature, all findings were correlational, limiting possibilities of causal inference.

In sum, the findings that sleep disturbances are associated with evidence of AD pathology and change over time in both amyloid and tau burden suggest an important correlate of AD pathogenesis. These findings may therefore be of interest when considering or designing sleep‐based interventions in AD prevention trials.

## AUTHOR CONTRIBUTIONS

Bery Mohammediyan had access to all the data in the study and takes full responsibility for the integrity of the data, and the accuracy of the analyses. Concept and design: Bery Mohammediyan, Andrée‐Ann Baril, and Sylvia Villeneuve. Acquisition, analysis or interpretation of data: All authors. Drafting of the manuscripts: Bery Mohammediyan, Andrée‐Ann Baril, and Sylvia Villeneuve. Critical revision of the manuscript for important intellectual content: Bery Mohammediyan, Andrée‐Ann Baril, John Breitner, and Sylvia Villeneuve. Statistical analysis: Bery Mohammediyan, Andrée‐Ann Baril, and Sylvia Villeneuve. Obtained funding: Sylvia Villeneuve. Administrative, technical, or material support: Bery Mohammediyan and Andrée‐Ann Baril. Supervision: Sylvia Villeneuve.

## CONFLICT OF INTEREST STATEMENT

A.‐A.B. received speaker fees from Eisai, outside of the scope of the present study. A.F.V. is supported by the CONACYT. S.D. has received research contracts from Biogen, Novo Nordisk, Passage Bio, Alnylam, Roche, and Eli Lilly; has served on speaker fees/advisory boards for Eisai, Eli Lilly, and Voyager Therapeutics; and is a member of the Data and Safety Monitoring Board (DSMB) for Aviado Bio. Industry partners played no role in the conduct of the research or the decision to publish. No other disclosures were reported. Author disclosures are available in the supporting information.

## CONSENT STATEMENT

All participants of the PREVENT‐AD cohort provided informed consent to participate in the study. The PREVENT‐AD has approval from the McGill Institutional Review Board and/or Douglas Mental Health University Institute Research Ethics Board.

## CODE AVAILABILITY STATEMENT

The code used for the statistical analyses is available from the first author upon request. The analyses and figures were performed in R 4.1.0, RStudio version 1.4.1717 for macOS, and we used the MASS package to conduct RLM. (Packages: svglite v2.1.0; tidyverse v1.3.2; dplyr v1.1.2; ggplot2 v3.4.2; MASS v7.3‐60.2; broom v1.0.3; sjPlot v2.8.12; robustlmm v2.3‐2; data.table v1.14.8; lubridate v1.9.2; Hmisc v5.2‐0; reshape2 v1.4.4).

## Supporting information



Supporting Information

Supporting Information

## Data Availability

Data used in this manuscript were obtained from the Pre‐symptomatic EValuation of Experimental or Novel Treatments for Alzheimer's Disease (PREVENT‐AD) program. The data of participants who agreed to open sharing are available to qualified researchers or physicians at https://registeredpreventad.loris.ca. The remaining data can be shared upon approval by the scientific committee at the Centre for Studies on Prevention of Alzheimer's Disease (StoP‐AD) at the Douglas Mental Health University Institute.

## 1

A list of all PREVENT‐AD collaborators can be found at the following website: https://preventad.loris.ca/acknowledgements/acknowledgements.php?date=2025‐10‐06&authors.
