# Supplementary material for: Longitudinal association between sleep and Alzheimer's pathology
Source: Alzheimers Dement. 2026 Mar 10;22(3):e71228. doi: 10.1002/alz.71228 (PMC12973143; doi:10.1002/alz.71228)
Supplement: Supplementary file 1 — Supporting Information [file ALZ-22-e71228-s001.docx]

**Supplemental Online Content**

**Longitudinal association between sleep and Alzheimer pathology**

Bery Mohammediyan^1,2^, MSc; Andrée-Ann Baril^1,2^, PhD; Alfonso Fajardo Valdez^3^, MSc; Frédéric St-Onge^3^, PhD; Alexa Pichet-Binette, PhD^4,5,6^; Julie Carrier^1,7^, PhD; Maiya R. Geddes, MD^3,8,9^; Simon Ducharme, MD^3,10^; Maxime Montembeault, PhD^3,8^; Jean-Paul Soucy^10^, MD;

John Breitner^3,8,10^, MD, MPH ; Judes Poirier^3,8^, PhD; Sylvia Villeneuve^3,8, 10^, PhD; for the PREVENT-AD Research Group

^1^Center for advanced research in sleep medicine, Research Center of the CIUSSS-NIM, Montreal, Quebec, Canada

^2^Department of Medicine, Université de Montréal, Montreal, Quebec, Canada

^3^Douglas Mental Health University Institute, Montreal, Quebec, Canada

^4^Centre de Recherche de l’Institut Universitaire de Gériatrie de Montréal, Montréal, Quebec, Canada

^5^Department of Physiologie and Pharmacology, Université de Montréal, Montréal, Quebec, Canada

^6^Clinical Memory Research Unit, Department of Clinical Sciences Malmö, Lund University, Lund, Sweden

^7^Department of Psychology, University of Montreal, Montreal, Quebec, Canada

^8^Department of Psychiatry, Faculty of Medicine, McGill University, Montreal, Quebec, Canada

^9^Massachusetts Institute of Technology, Cambridge, MA, USA

^10^McConnell Brain Imaging Centre, Montreal Neurological Institute, Montreal, Quebec, Canada

#### **Correspondence**

Sylvia Villeneuve, Douglas Mental Health University Institute, Centre for Studies on the Prevention of Alzheimer’s Disease (StoP-AD), Perry Pavilion Room E3417.1, 6875 Boulevard LaSalle, Montreal, QC H4H1R3, Canada. Email: sylvia.villeneuve@mcgill.ca

**Key words:** Sleep, PET, Amyloid, Tau, circadian rhythms

**Total word count of the main text in Supplement**: 168

**The supplementary includes** eMethods, 3 eTables, 2 efigures, 1 references

Mme. Mohammediyan bery.mohammediyan@mail.mcgill.ca

Dr. Baril andree-ann.baril@umontreal.ca

Mr. Fajardo Valdez alfonso.fajardovaldez@mail.mcgill.ca

Dr. St-Onge frederic.st-onge@mail.mcgill.ca

Dr. Carrier julie.carrier.1@umontreal.ca

Dr. Geddes maiya.geddes@mcgill.ca

Dr. Ducharme simon.ducharme@mcgill.ca

Dr. Montembeault maxime.montembeault@mcgill.ca

Dr. Soucy jean-paul.soucy@mcgill.ca

Dr. Breitner john.breitner@mcgill.ca

Dr. Poirier judes.poirier@mcgill.ca

**Table of contents**

Table S1. Longitudinal sample demographics
S1. Correlation matrix between subjective and objective sleep variables.

S2. Association between subjective sleep and tau meta-ROI SUVR restricted to one year after sleep assessment.

S3. Cross-sectional associations between baseline AD pathology measured with PET and average objective sleep measures.

S4. Longitudinal associations between AD pathology measured with PET and average objective sleep measures.

S5. Interaction between subjective and sleep variability, and amyloid status on longitudinal AD pathology measured with PET.

S6. Interaction average sleep measures, and amyloid status on longitudinal AD pathology measured with PET.

S7. Interaction between subjective sleep and objective sleep, and sex on AD pathology measured with PET.

S8. Interaction between average sleep measures, and sex on longitudinal AD pathology measured with PET.

S9. Interaction between subjective sleep and objective sleep, and sex on longitudinal AD pathology measured with PET.

S10. Interaction between average sleep measures, and sex on longitudinal AD pathology measured with PET.

S11. Interaction between subjective sleep and objective sleep, and APOE4 status on AD pathology measured with PET.

S12. Interaction between average sleep measures, and APOE4 status on AD pathology measured with PET.

S13. Interaction between subjective sleep and objective sleep, and APOE4 status on longitudinal AD pathology measured with PET.

S14. Interaction between average sleep measures, and APOE4 status on longitudinal AD pathology measured with PET.

**Supplementary Tables**

**Table S1. Longitudinal sample demographics**

|  | **Longitudinal AD pathology and PSQI (n=103)** | **Longitudinal AD pathology and Actigraphy (n=99)** |
| --- | --- | --- |
| Age (years) | 67.26 (4.89) | 67.31 (4.87) |
| Sex (%female) | 77 (71.96) | 76 (73.08) |
| Education (years) | 15.50 (39.23) | 15.34 (3.40) |
| BMI (kg/m2) | 26.71 (4.72) | 26.94 (5.26) |
| Retirement (%) | 72 (67.29) | 71 (68.27) |
| APOE4 status (%positive) | 42 (39.25) | 40 (38.46) |
| **Sleep characteristics (PSQI)** |  |  |
| PSQI baseline | 6.07 (3.55) | 6.04 (3.61) |
| **Sleep characteristics (average; actigraphy)** |  |  |
| Sleep duration (min) | 431.28 (49.24) | 430.94 (48.87) |
| Sleep efficiency (%) | 87.07 (5.50) | 86.92 (5.69) |
| Sleep fragmentation | 11.74 (4.92) | 11.93 (5.07) |
| **Sleep characteristics (day-to-day variability; actigraphy)** |  |  |
| Sleep duration (min) | 58.29 (28.96) | 58.18 (28.55) |
| Sleep efficiency (%) | 5.52 (4.45) | 5.50 (4.39) |
| Sleep fragmentation | 4.15 (2.04) | 4.15 (2.03) |
| **AD pathology** |  |  |
| Amyloid | 1.31 (0.31) | 1.29 (0.29) |
| Tau | 1.15 (0.11) | 1.15 (0.10) |
| **AD pathology slope** |  |  |
| Amyloid slope | 0.02 (0.03) | 0.02 (0.03) |
| Tau slope | ^a^0.003 (0.02) | ^b^0.003 (0.02) |

Baseline and longitudinal data are presented as n (%) for categorical variables, and as mean ± SD for continuous variables. Age, education, BMI, and retirement status are shown at baseline. AD pathology annual change (available for 103 individuals who also have a baseline PSQI and 99 individuals who also have baseline actigraphy) The demographics, pathological, and sleep characteristics of both subsamples were quite similar. Amyloid and tau data represent the mean standardized uptake value ratio (SUVR) at baseline. The mean between the two PET scans was 4.33 ± 0.53 y, range: 1.59 – 6.11 y. ^a^Longitudinal tau data was available for 97 participants. ^b^Longitudinal tau data was available for 93 participants. Abbreviations: BMI = body mass index; APOE = apolipoprotein E; PSQI = Pittsburgh Sleep Quality Index; AD = Alzheimer’s Disease

**Supplementary Figures**

** S1. Correlation matrix between subjective and objective sleep variables.** 186 participants had both subjective and objective baseline sleep data. The matrix shows the correlation coefficient between subjective (PSQI) and objective (day-to-day variability or average sleep measures extracted from 7-day actigraphy data collection) sleep variables. The value in each matrix square represents the correlation coefficient between the two corresponding variables (Pearson’s r). Darker orange colors represent stronger positive correlations whereas darker red colors represent stronger negative correlations. Gray squares on the diagonal indicate self-correlations, which are always equal to 1. * p_uncorrected_<0.05; ** p_uncorrected_ < 0.01.

**S2. Association between subjective sleep and tau meta-ROI SUVR restricted to one year after sleep assessment.** Age at baseline PET scan, sex, BMI and time between predictor (PSQI) and the outcome (tau-PET) were accounted for. These figures shows the association between PSQI global score and baseline global amyloid (n=68) or tau meta-ROI (n=67) SUVR measured with PET including only individuals whose sleep assessment was within one year of their PET scan. Gray shading represents the 95% confidence interval. Results in **bold** are significant p_uncorrected_ <0.05. Results with a star (*) survive FDR corrections. Abbreviations: PSQI = Pittsburgh Sleep Quality Index; Aβ = amyloid-beta; SUVR= Standardized uptake value ratio; ROI= Region of interest; β= standardized coefficient; CI = standardized Confidence Interval

**S3. Cross-sectional associations between baseline AD pathology measured with PET and average objective sleep measures.** Age at baseline PET scan, sex, BMI and time between predictor (sleep average) and the outcome (amyloid or tau-PET) were accounted for in every model. A) The matrix shows the association between a specified sleep variable (averaged sleep measures extracted from 7-day actigraphy data collection) and the baseline AD PET. The matrix’s color grading shows significant associations where lighter colors are more significant and darker colors are closer to p_uncorrected_ =0.05. Gray squares show non-significant associations. B-I) show the specific scatterplots of these associations. Gray shading represents the 95% confidence interval. Results in **bold** are significant p_uncorrected_ <0.05. Results with a star (*) survive FDR corrections. Abbreviations: PSQI = Pittsburgh Sleep Quality Index; SUVR = Standardized uptake value ratio; Aβ = amyloid-beta; ROI = Region of interest; β= standardized coefficient; CI = standardized Confidence Interval

**S4. Longitudinal associations between AD pathology measured with PET and average objective sleep measures.** Age at baseline PET scan, sex, and BMI were accounted for in every model. A) The matrix shows the association between a specified sleep variable (averaged sleep measures extracted from 7-day actigraphy data collection) and longitudinal AD PET. The matrix’s color grading shows significant associations where lighter colors are more significant and darker colors are closer to p_uncorrected_ =0.05. Gray squares show non-significant associations. B-I) show the specific scatterplots of these associations. In all models, longitudinal AD pathology is measured by extracting the annual slope of SUVR accumulation for amyloid and tau accumulation, respectively. In all the graphs gray shading represents the 95% confidence interval. PSQI = Pittsburgh Sleep Quality Index; SUVR = Standardized uptake value ratio; Aβ = amyloid-beta; ROI = Region of interest; β= standardized coefficient; CI = standardized Confidence Interval

**S5. Interaction between subjective and sleep variability, and amyloid status on longitudinal AD pathology measured with PET.** Age at baseline PET scan, sex, and BMI were accounted for in every model. Baseline amyloid status was categorized into two groups based on global SUVR: Group A- (gray): SUVR < 1.26, Group A+ (orange): SUVR ≥ 1.26. These figures show longitudinal associations between global Aβ or tau meta-ROI SUVR slope and the interaction of each sleep variable (PSQI global score or actigraphy-derived sleep variability) with baseline Aβ status. Interaction statistics are reported for each models. Shaded regions (gray, or orange) represent 95% confidence interval. Results in **bold** are significant p_uncorrected_ <0.05. Results with a star (*) survive FDR corrections. Abbreviations: PSQI = Pittsburgh Sleep Quality Index; Aβ = amyloid-beta; SUVR= Standardized uptake value ratio; ROI= Region of interest; β= standardized coefficient; CI = standardized Confidence Interval

**S6. Interaction average sleep measures, and amyloid status on longitudinal AD pathology measured with PET.** Age at baseline PET scan, sex, and BMI were accounted for in every model. Baseline amyloid status was categorized into two groups based on global SUVR: Group A- (gray): SUVR < 1.26, Group A+ (orange): SUVR ≥ 1.26. These figures show longitudinal associations between global Aβ or tau meta-ROI SUVR slope and the interaction of each sleep variable (average actigraphy-derived sleep measures) with baseline Aβ status. Interaction statistics are reported for each models. Shaded regions (gray, or orange) represent 95% confidence interval. Results in **bold** are significant p_uncorrected_ <0.05. Results with a star (*) survive FDR corrections. Abbreviations: PSQI = Pittsburgh Sleep Quality Index; Aβ = amyloid-beta; SUVR= Standardized uptake value ratio; ROI= Region of interest; β= standardized coefficient; CI = standardized Confidence Interval

**S7. Interaction between subjective sleep and objective sleep, and sex on AD pathology measured with PET.** Age at baseline PET scan, BMI and time between predictor (PSQI or sleep variability) and the outcome (amyloid or tau-PET) were accounted for in every model. In all models, females are shown in gray and males in red. These figures show cross-sectional associations between the interaction of each sleep variable (PSQI global score, or actigraphy-derived sleep variability) and sex with AD pathology, represented by baseline global Aβ or tau meta-ROI SUVR. Interaction statistics are reported for each model. Shaded regions (gray, or red) represent 95% confidence interval. Abbreviations: PSQI = Pittsburgh Sleep Quality Index; Aβ = amyloid-beta; SUVR= Standardized uptake value ratio; ROI= Region of interest; β= standardized coefficient; CI = standardized Confidence Interval

**S8. Interaction between average sleep measures, and sex on longitudinal AD pathology measured with PET.** Age at baseline PET scan, BMI and time between predictor (average sleep measures) and the outcome (amyloid or tau-PET) were accounted for in every model. In all models, females are shown in gray and males in red. These figures show cross-sectional associations between the interaction of each sleep variable (average actigraphy-derived sleep measures), and sex on AD pathology, represented by baseline global Aβ or tau meta-ROI SUVR. Interaction statistics are reported for each model. Shaded regions (gray, or red) represent 95% confidence interval. Abbreviations: PSQI = Pittsburgh Sleep Quality Index; Aβ = amyloid-beta; SUVR= Standardized uptake value ratio; ROI= Region of interest; β= standardized coefficient; CI = standardized Confidence Interval

**S9. Interaction between subjective sleep and objective sleep, and sex on longitudinal AD pathology measured with PET.** Age at baseline PET scan, and BMI were accounted for in every model. In all models, females are shown in gray and males in red. These figures show longitudinal associations between the interaction of each sleep variable (PSQI global score, or actigraphy-derived sleep variability) and sex with AD pathology, represented by longitudinal global Aβ or tau meta-ROI SUVR slope. Interaction statistics are reported for each model. Shaded regions (gray, or red) represent 95% confidence interval. Abbreviations: PSQI = Pittsburgh Sleep Quality Index; Aβ = amyloid-beta; SUVR= Standardized uptake value ratio; ROI= Region of interest; β= standardized coefficient; CI = standardized Confidence Interval

**S10. Interaction between average sleep measures, and sex on longitudinal AD pathology measured with PET.** Age at baseline PET scan, and BMI were accounted for in every model. In all models, females are shown in gray and males in red. These figures show longitudinal associations between the interaction of each sleep variable (average actigraphy-derived sleep measures) and sex with AD pathology, represented by longitudinal global Aβ or tau meta-ROI SUVR slope. Interaction statistics are reported for each model. Shaded regions (gray, or red) represent 95% confidence interval. Results in **bold** are significant p_uncorrected_ <0.05. Results with a star (*) survive FDR corrections. Abbreviations: PSQI = Pittsburgh Sleep Quality Index; Aβ = amyloid-beta; SUVR= Standardized uptake value ratio; ROI= Region of interest; β= standardized coefficient; CI = standardized Confidence Interval

**S11. Interaction between subjective sleep and objective sleep, and APOE4 status on AD pathology measured with PET.** Age at baseline PET scan, sex, BMI and time between predictor (PSQI or sleep variability) and the outcome (amyloid or tau-PET) were accounted for in every model. In all models, individuals with no APOE ε4 allele are shown in gray and individuals with at least one APOE ε4 allele are shown in yellow. These figures show cross-sectional associations between the interaction of each sleep variable (PSQI global score, or actigraphy-derived sleep variability) and APOE4 status with AD pathology, represented by baseline global Aβ or tau meta-ROI SUVR. Interaction statistics are reported for each model. Shaded regions (gray, or yellow) represent 95% confidence interval. Abbreviations: PSQI = Pittsburgh Sleep Quality Index; Aβ = amyloid-beta; SUVR= Standardized uptake value ratio; ROI= Region of interest; APOE = apolipoprotein E; β= standardized coefficient; CI = standardized Confidence Interval

**S12. Interaction between average sleep measures, and APOE4 status on AD pathology measured with PET.** Age at baseline PET scan, sex, BMI and time between predictor (average sleep measures) and the outcome (amyloid or tau-PET) were accounted for in every model. In all models, individuals with no APOE ε4 allele are shown in gray and individuals with at least one APOE ε4 allele are shown in yellow. These figures show cross-sectional associations between the interaction of each sleep variable (average actigraphy-derived sleep measures) and APOE4 status with AD pathology, represented by baseline global Aβ or tau meta-ROI SUVR. Interaction statistics are reported for each model. Shaded regions (gray, or yellow) represent 95% confidence interval. Abbreviations: PSQI = Pittsburgh Sleep Quality Index; Aβ = amyloid-beta; SUVR= Standardized uptake value ratio; ROI= Region of interest; APOE = apolipoprotein E; β= standardized coefficient; CI = standardized Confidence Interval

**S13. Interaction between subjective sleep and objective sleep, and APOE4 status on longitudinal AD pathology measured with PET.** Age at baseline PET scan, sex, and BMI were accounted for in every model. In all models, individuals with no APOE ε4 allele are shown in gray and individuals with at least one APOE ε4 allele are shown in yellow. These figures show longitudinal associations between the interaction of each sleep variable (PSQI global score, or actigraphy-derived sleep variability) and APOE4 status with longitudinal AD pathology, represented by baseline global Aβ or tau meta-ROI SUVR slope. Interaction statistics are reported for each model. Shaded regions (gray, or yellow) represent 95% confidence interval. Abbreviations: PSQI = Pittsburgh Sleep Quality Index; Aβ = amyloid-beta; SUVR= Standardized uptake value ratio; ROI= Region of interest; APOE = apolipoprotein E; β= standardized coefficient; CI = standardized Confidence Interval

**S14. Interaction between average sleep measures, and APOE4 status on longitudinal AD pathology measured with PET.** Age at baseline PET scan, sex, and BMI were accounted for in every model. In all models, individuals with no APOE ε4 allele are shown in gray and individuals with at least one APOE ε4 allele are shown in yellow. These figures show longitudinal associations between the interaction of each sleep variable (average actigraphy-derived sleep measures) and APOE4 status with longitudinal AD pathology, represented by baseline global Aβ or tau meta-ROI SUVR slope. Interaction statistics are reported for each model. Results in **bold** are significant p_uncorrected_ <0.05. Results with a star (*) survive FDR corrections. Abbreviations: PSQI = Pittsburgh Sleep Quality Index; Aβ = amyloid-beta; SUVR= Standardized uptake value ratio; ROI= Region of interest; APOE = apolipoprotein E; β= standardized coefficient; CI = standardized Confidence Interval
